# Commentary: Impact of HPV vaccine on CIN2+ recurrence after conization: a systematic review and meta-analysis of vaccination timing, valency and surgical margins

**DOI:** 10.3389/fonc.2026.1914736

**Published:** 2026-08-24

**Authors:** Mengjie Xue, Jihong Peng

**Affiliations:** Department of Obstetrics and Gynecology, Liyang Branch of Jiangsu Hospital of Traditional Chinese Medicine, Liyang, Jiangsu, China

**Keywords:** cervical conization, CIN2+ recurrence, healthy-user bias, HPV vaccination, meta-analysis, randomized controlled trial

## 1 Introduction

Maiorano et al. recently published a comprehensive systematic review and meta-analysis in this journal examining the impact of adjuvant HPV vaccination on CIN2+ recurrence after conization (1). Across 17 studies and 33,181 women, they reported a pooled relative risk of 0.38 (95% CI 0.29-0.51), representing approximately a 62% reduction in recurrence risk. This is an important and timely synthesis that has clear implications for clinical practice.

We wish to draw attention to one aspect of these results that we believe merits additional discussion: the influence of study design on the pooled estimate. Fifteen of the 17 included studies employed observational designs. The concept of healthy user bias is particularly relevant in this context: women who choose to receive HPV vaccination after conization may be more health-conscious and more likely to adhere to follow-up screening — a confounding factor that observational studies cannot fully control for. Maiorano et al.’s own meta-regression found that study design explained 40.7% of between-study heterogeneity, with an estimated RR of approximately 0.29 for observational studies versus 0.55 for randomised trials. The trim-and-fill corrected estimate of 0.50 further reinforces the possibility that unadjusted observational data may overstate the magnitude of benefit.

## 2 Re-analysis restricted to randomised evidence

To obtain an estimate derived exclusively from randomised data, we pooled the four RCTs included in Maiorano et al.’s Table 2: Hildesheim et al. (2), Pieralli et al. (3), Karimi-Zarchi et al. (4), and van de Laar et al. (5). Data were independently extracted by two authors (MX and JP) using a pre-specified form. No additional eligibility criteria beyond those in Maiorano et al. were applied. As this is a reanalysis of published data, no new literature search was conducted. A DerSimonian-Laird random-effects model with a 0.5 continuity correction was applied to account for heterogeneity and to include the Pieralli trial (zero events in the vaccinated arm). We acknowledge that this correction is conservative and may bias the estimate toward the null. As sensitivity analyses, we also computed the Peto odds ratio (OR 0.52; 95% CI 0.32–0.84) and we performed a leave-one-out sensitivity analysis (**Table 1**). Both authors independently ran the main analysis in RevMan 5.4 and obtained identical results.

**Table 1:** Leave-one-out sensitivity analysis.

Excluded study	RR	95% CI
None (all 4 trials)	0.54	0.39–0.74
Hildesheim (2)	0.52	0.38–0.72
Pieralli (3)	0.55	0.40–0.76
Karimi-Zarchi (4)	0.68	0.43–1.10
van de Laar (5)	0.47	0.31–0.71

**Figure 1:**
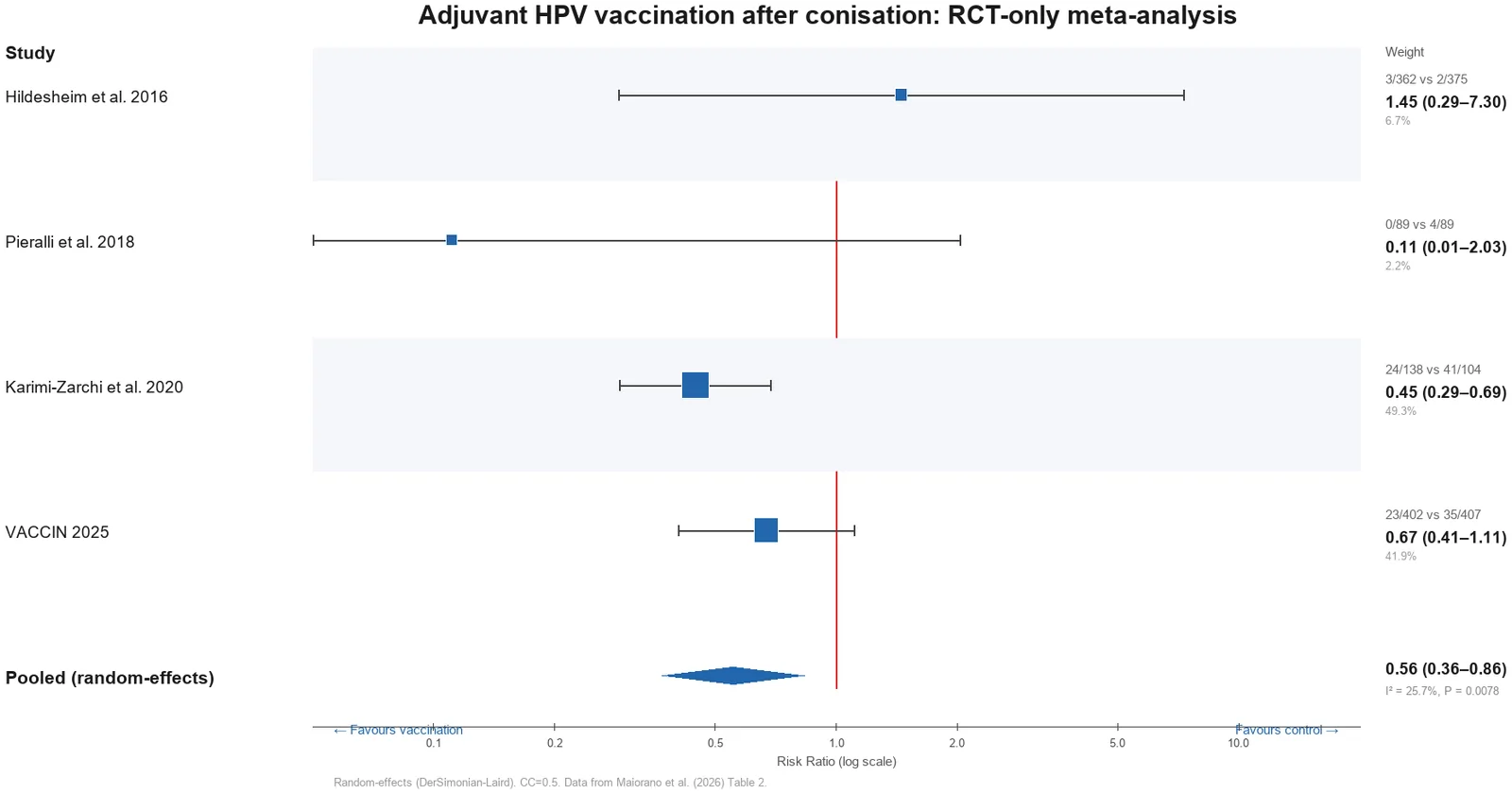
Forest plot of the four available randomised controlled trials of adjuvant HPV vaccination after conization, using data from Maiorano et al. (1) Table 2. Pooled random-effects estimate: RR 0.56 (95% CI 0.36-0.86; P = 0.008; I^2^ = 25.7%). Weights are from random-effects analysis.

A DerSimonian-Laird random-effects model with continuity correction of 0.5 yielded a pooled RR of 0.56 (95% CI 0.36-0.86; 95% prediction interval 0.28-1.12; P = 0.008; I2 = 25.7%; **Figure 1**). This corresponds to a 44% reduction in recurrence. The wide prediction interval reflects the limited number of trials and the uncertainty in estimating the range of true effects in future populations. Leave-one-out analysis confirmed robustness, with all point estimates favoring vaccination (**Table 1**).

We recognise the limitations inherent in this re-analysis. Three of the four trials were open-label, and the total number of events is modest. The Hildesheim trial showed a null-to-negative effect (RR 1.10; 95% CI 0.23-5.29), and the Karimi-Zarchi trial was noted in the original meta-analysis to have potential bias concerns related to allocation concealment and blinding. The inclusion of these underpowered and methodologically heterogeneous trials tempers the precision of the pooled estimate. However, the direction of effect is consistent across all four trials. The difference between the observational and randomised estimates is consistent with, but does not prove, healthy-user bias. Even with these limitations, the pattern across multiple analytical approaches (RCT-only pooling, meta-regression, trim-and-fill, and leave-one-out sensitivity analysis) is consistent in suggesting a more modest treatment effect than the unadjusted pooled RR of 0.38 implies.

## 3 Discussion

This does not contradict the overall conclusion of Maiorano et al. that vaccination is beneficial (6); indeed, we recognise that Maiorano et al. themselves acknowledged the potential for overestimation in their discussion and provided subgroup analyses by study design. Our reanalysis refines, rather than refutes, their findings. The question is one of magnitude. A 44% reduction in CIN2+ recurrence is clinically important. However, the distinction between a 44% and a 62% reduction has practical implications for cost-effectiveness modelling, particularly in settings where health resources are constrained. Guideline panels currently present a single pooled estimate without disaggregating by study design, which may convey greater precision and a larger effect than the data support (7).

We strongly recommend that future syntheses and clinical guidance documents present estimates stratified by study design, or at minimum discuss the range of plausible effect sizes rather than a single number. Individual patient data (IPD) meta-analyses should be pursued as the gold standard for quantifying the effect of adjuvant HPV vaccination on post-conization recurrence. A large, confirmatory RCT with CIN2+ recurrence as the primary endpoint would substantially reduce the remaining uncertainty.

A final note on interpretation. The CI of our pooled estimate (0.36-0.86) overlaps with that of the observational estimate (0.29-0.51). Our analysis does not rule out an effect as large as the observational data suggest. What gives us pause is that three distinct approaches (meta-regression, trim-and-fill correction, and RCT-only pooling) all pull the point estimate away from 0.38 and toward 0.50-0.56. That consistency, more than any single number, is worth keeping in mind when the evidence is translated into guidelines.
